# Emergence of an Outbreak-Associated Clostridium difficile Variant with Increased Virulence

**DOI:** 10.1128/JCM.03058-14

**Published:** 2015-03-18

**Authors:** Carlos Quesada-Gómez, Diana López-Ureña, Luis Acuña-Amador, Manuel Villalobos-Zúñiga, Tim Du, Rosemayre Freire, Caterina Guzmán-Verri, María del Mar Gamboa-Coronado, Trevor D. Lawley, Edgardo Moreno, Michael R. Mulvey, Gerly Anne de Castro Brito, Evelyn Rodríguez-Cavallini, César Rodríguez, Esteban Chaves-Olarte

**Affiliations:** a Facultad de Microbiología and Centro de Investigación en Enfermedades Tropicales, Universidad de Costa Rica, San José, Costa Rica; b Hospital San Juan de Dios, CCSS, San José, Costa Rica; c National Microbiology Laboratory, Public Health Agency of Canada, Winnipeg, Manitoba, Canada; d Departamento de Morfologia, Universidade Federal do Ceará, Fortaleza, Brazil; e Programa de Investigación en Enfermedades Tropicales, Escuela de Medicina Veterinaria, Universidad Nacional, Heredia, Costa Rica; f Host-Microbiota Interactions Laboratory, Wellcome Trust Sanger Institute, Hinxton, United Kingdom; g Instituto Clodomiro Picado, Universidad de Costa Rica, San José, Costa Rica

## Abstract

The prevalence of Clostridium difficile infections has increased due to the emergence of epidemic variants from diverse genetic lineages. Here we describe the emergence of a novel variant during an outbreak in a Costa Rican hospital that was associated with severe clinical presentations. This C. difficile variant elicited higher white blood cell counts and caused disease in younger patients than did other strains isolated during the outbreak. Furthermore, it had a recurrence rate, a 30-day attributable disease rate, and disease severity as great as those of the epidemic strain NAP1. Pulsed-field gel electrophoresis genotyping indicated that the outbreak strains belong to a previously undescribed variant, designated NAP_CR1_. Whole-genome sequencing and ribotyping indicated that the NAP_CR1_ variant belongs to C. difficile ribotype 012 and sequence type 54, as does the reference strain 630. NAP_CR1_ strains are resistant to fluoroquinolones due to a mutation in *gyrA*, and they possess an 18-bp deletion in *tcdC* that is characteristic of the epidemic, evolutionarily distinct, C. difficile NAP1 variant. NAP_CR1_ genomes contain 10% more predicted genes than strain 630, most of which are of hypothetical function and are present on phages and other mobile genetic elements. The increased virulence of NAP_CR1_ was confirmed by mortality rates in the hamster model and strong inflammatory responses induced by bacteria-free supernatants in the murine ligated loop model. However, NAP_CR1_ strains do not synthesize toxin A and toxin B at levels comparable to those in NAP1 strains. Our results suggest that the pathogenic potential of this emerging C. difficile variant is due to the acquisition of hypothetical functions associated with laterally acquired DNA.

## INTRODUCTION

Clostridium difficile is a Gram-positive, anaerobic, spore-forming bacillus recognized as a common source of health care infections (1). Antibiotic treatment suppresses the intestinal microbiota, allowing colonization and germination of C. difficile spores. After colonization, the bacterium produces two exotoxins that glucosylate monomeric GTPases, i.e., toxin A (TcdA) and toxin B (TcdB). Their action results in the characteristic pathology of C. difficile infections (CDIs), ranging from mild diarrhea to severe pseudomembranous colitis.

Since 2003, highly virulent toxigenic C. difficile strains have caused epidemics characterized by greater incidence, severity, and fatality of disease (2). These strains, initially classified as “hypervirulent,” cluster into a distinct phylogenetic group (3), being classified as group BI (restriction endonuclease analysis [REA]), type NAP1 (pulsed-field gel electrophoresis [PFGE]), ribotype 027 (PCR ribotyping), and toxinotype III (toxin gene polymorphism typing) (4). NAP1 strains have spread widely in recent years. These strains have been responsible for severe epidemic outbreaks throughout the world (2, 5, 6) and have been implicated in the severe outcomes of C. difficile infections (7).

NAP1 strains produce a binary toxin (C. difficile binary toxin [CDT]) and harbor a point mutation in the *tcdC* gene, which encodes a putative negative transcriptional regulator of C. difficile toxins. It is postulated that the truncated TcdC is unable to downregulate *tcdA* and *tcdB* transcription, resulting in increased toxin production (8). Several studies have attributed the hypervirulence of NAP1 strains to this trait (8, 9). However, other lines of evidence indicate that *tcdC* truncations and disease severity are not related (10, 11). Furthermore, the association between increased *in vitro* toxin production and strains with high virulence is also controversial. Akerlund and collaborators (12) noted a correlation between disease severity and toxin concentrations in feces, but there was no relationship between levels of toxin synthesized *in vitro* by a group of NAP1 strains and fecal toxin levels (12).

The prevalence and severity of human infections caused by strains different from NAP1 are increasing (7, 13–16). For instance, NAP7 (ribotype 078) strains have been associated with severe disease in younger populations and have been isolated in cases of community-associated CDIs (17). The clinical spectrum induced by these NAP7 strains indicates that they might represent an emerging epidemic genotype; however, the molecular determinants associated with this behavior have not been addressed as thoroughly as for NAP1 strains. Other strains associated with severe disease have been recently described as well (18). In 2009 to 2010, a C. difficile outbreak occurred in a tertiary care hospital in Costa Rica. In a preliminary study performed with a partial collection of isolates from this outbreak, the presence of the NAP1 genotype was reported (19). Interestingly, a group of fluoroquinolone-resistant strains without NAP designation were also isolated (19). In this work, we report a group of C. difficile strains belonging to a previously undescribed NAP type with pathogenic potential similar to that of epidemic NAP1 strains. This emerging genotype is highly resistant to fluoroquinolones and possesses a deletion in *tcdC* similar to NAP1 strains; however, it lacks CDT and does not produce increased amounts of TcdA and TcdB. Together, these results describe the emergence of a C. difficile variant with high virulence potential.

## MATERIALS AND METHODS

### C. difficile isolation and strains.

Stool samples positive for C. difficile toxins (Xpect Clostridium difficile toxin A/B test; Oxoid, Basingstoke, United Kingdom) that were collected during a CDI outbreak were processed. Samples were treated with 96% ethanol and inoculated onto cefoxitin-cycloserine-fructose agar (CCFA) plates (Oxoid, Basingstoke, United Kingdom), which were incubated for 5 days in an anaerobic chamber (Bactron II; Shel Lab, Cornelius, OR) under an atmosphere of 90% N_2_, 5% H_2_, and 5% CO_2_. Colonies were identified phenotypically (RapID 32A system; bioMérieux, Marcy l'Etoile, France), chemotaxonomically (Sherlock fatty acid methyl ester [FAME] analysis system; MIDI, Santa Clara, CA), and by PCR amplification of the *tpi* gene (20).

### PCR-based genotyping.

DNA from each clinical isolate was obtained from overnight cultures in brain heart infusion (BHI) broth (Oxoid), using the InstaGene reagent (Bio-Rad, Hercules, CA). Fragments of *tcdA*, *tcdB*, *cdtB*, and *tcdC* were amplified by PCR using primers and conditions reported previously (21).

### Pulsed-field gel electrophoresis.

The PFGE procedure used was derived from published protocols (4, 22). Briefly, bacteria from 6- to 8-h cultures in BHI broth were disrupted in lysis buffer. Agarose plugs were prepared by mixing equal volumes of bacterial suspensions and Seakem Gold agarose (Lonza, Basel, Switzerland) in 1× Tris-EDTA (TE) buffer (Sigma, Deisenhofen, Germany) containing SDS (Sigma). The plugs were incubated in a buffer composed of lysozyme, RNase A, and mutanolysin (Sigma). After overnight digestion with SmaI (Roche, Mannheim, Germany), DNA fragments were separated on 1% agarose gels in 0.5× Tris-borate-EDTA (TBE) buffer (Fermentas, St-Leon-Rot, Germany) containing 50 μM thiourea (Sigma), using a CHEF-DRII system (Bio-Rad). Images were analyzed with BioNumerics software (version 5.1; Applied Maths, Austin, TX) and macrorestriction patterns were compared to those deposited in the database of the National Microbiology Laboratory of the Public Health Agency of Canada (Winnipeg, Canada).

### Antimicrobial susceptibility testing.

MICs for ciprofloxacin, moxifloxacin, levofloxacin, clindamycin, metronidazole, rifampin, and vancomycin (Sigma) were determined using agar dilution, following the guidelines of the Clinical and Laboratory Standards Institute (23). Resistance breakpoints were set as follows: ciprofloxacin, >4 μg/ml; moxifloxacin, >4 μg/ml; levofloxacin, >4 μg/ml; clindamycin, >4 μg/ml; metronidazole, >16 μg/ml; rifampin, >32 μg/ml.

### Clinical data.

The study was a retrospective cohort study with patients with positive and confirmed C. difficile stool cultures. Each case was classified as nosocomial CDI or community-associated CDI according to criteria from the Infectious Diseases Society of America (IDSA) (24). CDI severity was categorized by applying the IDSA/Society for Healthcare Epidemiology of America (SHEA) criteria (24, 25) and criteria described by Zar et al. (26). Clinical data were extracted from patients' medical records (27). The 30-day attributable mortality rate was calculated by considering patients with positive and confirmed C. difficile stool cultures who presented clinical signs and symptoms of CDI (temperature above 38°C, white blood cell count above 15,000 cell/mm^3^, or radiological evidence of pseudomembranous colitis) and whose death occurred within 30 days after the first diarrheal discharge. Categorical variables were analyzed by using logistic regression models, and risk factors were expressed in terms of odds ratios (ORs) with 95% confidence intervals (CIs). Two-tailed *P* values of 0.05 were used for significance. All statistical analyses were performed using IBM SPSS Statistics 20 software (IBM, Armonk, NY). Data collection was approved by the Ethics Committee of the San Juan de Dios Hospital (protocol CLOBI-HSJD-018-2009).

### Whole-genome sequencing and sequence analysis.

Whole-genome sequences of representative strains of each of the four NAP_CR1_ subtypes were obtained using multiplexed paired-end libraries and the sequencing-by-synthesis Illumina HiSeq platform. To this end, reads were assembled using Velvet (28), and contigs of >300 bp were scaffolded with SSPACE (29) and ordered with ABACAS, using the C. difficile strain 630 genome as the reference (30). Gaps were filled using GapFiller (31), and the reads were mapped back to the assembly using SMALT (http://www.sanger.ac.uk/resources/software/smalt). Single-nucleotide polymorphisms (SNPs) were identified with RealPhy (version 1.07) (32) or with SAMTools (33). A dendrogram based on core SNPs was inferred via PhyML (34) and depicted using FigTree (http://tree.bio.ed.ac.uk/software/figtree). Deletions or truncations in *tcdC*, as well as mutations in *gyrA* and *gyrB* known to confer fluoroquinolone resistance, were identified using Artemis (35) and BLAST. Average nucleotide identities (ANIs) were computed using the genome-to-genome distance calculator at the German Collection of Microorganisms and Cell Cultures (36), and comparative genomic analyses were performed using the RAST server of the SEED framework (37).

### Multilocus sequence typing and ribotyping.

The sequence types (STs) of representative strains for each of the four NAP_CR1_ PFGE patterns were determined by using the multilocus sequence typing (MLST) 1.7 server maintained by the Center for Genomic Epidemiology at the Danish Technical University (38) and the classification scheme based on the genes *adk*, *atpA*, *tpi*, *glyA*, *dxr*, *sodA*, and *recA*, as proposed by Griffiths et al. (39). For ribotyping, primer sequences and reaction conditions were taken from the report by Bidet et al. (40).

### Hamster infection model.

For the animal models, one representative strain was selected from each group isolated during the outbreak. All of the strains tested were resistant to clindamycin (NAP_CR1_ MIC, 256 μg/ml; NAP1 MIC, 16 μg/ml; NAP4 MIC, 8 μg/ml). Groups of 5 adult female Syrian Golden hamsters (150 to 180 g) were treated subcutaneously with 10 mg/kg clindamycin phosphate on day −2. On day 0, clindamycin-treated and nontreated control hamsters were inoculated, through the orogastric route, with 1,000 spores of the outbreak strains or the nontoxigenic ATCC 700057 strain resuspended in Dulbecco's modified Eagle medium (DMEM) (Sigma) (41). Hamsters were monitored at 12-h intervals for signs of C. difficile infection, such as diarrhea, and death. On days 1, 6, and 12, fecal pellets and intestinal contents of dead and surviving animals were processed for C. difficile isolation (42), and the resulting isolates were typed by PFGE to confirm the identity of the inoculated strain. All animal experiments were approved by the Animal Care and Use Committee of the Universidad de Costa Rica (protocols CICUA 01-12 and CICUA 07-13).

### Murine ileal loop model.

The strains were grown in TYT medium (3% Bacto tryptose, 2% yeast extract, and 0.1% thioglycolate [pH 6.8]) (Sigma) for the indicated times. Bacteria were removed by centrifugation at 20,000 × *g* for 30 min, and supernatants were passed through 0.2-μm filters.

Male Swiss mice (20 to 25 g) were fasted overnight and anesthetized with ketamine (60 mg/kg) and xylazine (5 mg/kg) (König, São Paulo, Brazil). Through a midline laparotomy, a 4-cm ileal loop was ligated and injected with 0.3 ml of supernatants or the corresponding control solutions. Mice were sacrificed 4 h after inoculation, and the length and weight of the intestinal loops were recorded (43). Intestinal sections were fixed in formalin and stained with hematoxylin and eosin for histopathological evaluation. The samples were evaluated for the severity of epithelial damage, edema, and neutrophil infiltration using a histopathological score (HS) scale ranging from 0 (absence of alterations) to 3 (severe) (44). The neutrophil accumulation in homogenized ileal tissue was evaluated through determination of myeloperoxidase (MPO) activity with an assay using *o*-dianisidine dihydrochloride (Sigma) and H_2_O_2_ (45); the results were expressed as units of MPO/100 mg of ileal tissue. The concentrations of the proinflammatory cytokines interleukin 1β (IL-1β), IL-6, and tumor necrosis factor alpha (TNF-α) in ileal tissue homogenates were determined by commercial enzyme-linked immunosorbent assay (ELISA), following the instructions of the manufacturer (R&D Systems, Minneapolis, MN).

### Cytotoxicity assays.

Ten-fold dilutions of the supernatants were added to HeLa cell monolayers grown in DMEM supplemented with 5% fetal bovine serum. The cells were monitored for the appearance of cytopathic effect (CPE) by optical microscopy. TcdB-specific antiserum (TechLab, Blacksburg, VA) was used to neutralize the effect of the toxin. Cytotoxicity was expressed as the inverse of the dilution of the supernatants that caused 50% cell rounding in the monolayers (i.e., 50% CPE [CPE_50_]).

### Toxin quantitation.

The toxins were quantified in the same strains as used for the animal models. The amounts of toxins secreted by the strains were quantified by Western blotting. Proteins from bacteria-free supernatants were concentrated by methanol-chloroform precipitation. Proteins were separated in 7.5% SDS-PAGE gels and electrotransferred to polyvinylidene difluoride (PVDF) membranes. These membranes were probed with monoclonal anti-TcdA (TTC8) or anti-TcdB (2CV) antibodies (tgcBIOMICS, Mainz, Germany) (46). Chemiluminescence signals emitted after addition of a goat anti-mouse IgG-horseradish peroxidase conjugate (Invitrogen; Life Technologies, Carlsbad, CA) and the Lumi-Light Plus Western blotting substrate (Roche) were recorded with a Chemidoc XRS documentation system (Bio-Rad).

Transcripts of *tcdA* and *tcdB* were quantified by quantitative reverse transcription (qRT)-PCR. The different strains were grown on TYT medium, and 1 × 10^9^ cells were processed for RNA extraction. Bacteria were pelleted by centrifugation at 5,000 × *g* and lysed with lysostaphin (Sigma), acetic acid, proteinase K (Fermentas; Fisher Scientific, Pittsburgh, PA), and SDS (Sigma) (47). RNA was isolated with the RNeasy Midi kit (Qiagen, Hilden, Germany) and treated with DNase I Turbo (Ambion; Life Technologies, Austin, TX). Two micrograms of RNA was reverse transcribed to cDNA using RevertAid transcriptase (Fermentas). The amplification conditions were as reported previously (48). The relative expression of genes was calculated with the threshold cycle (ΔΔ*C_T_*) method, using the *rpoA* transcript as an endogenous control.

### Nucleotide sequence accession numbers.

The sequence information from this whole-genome shotgun project has been submitted to DDBJ/EMBL/GenBank under accession numbers JXCP00000000, JXBP00000000, JXBQ00000000, JXBR00000000, and JXBS00000000, as part of BioProject PRJNA264745.

## RESULTS

### Isolation and molecular characterization of an emerging strain.

**FIG 1:**
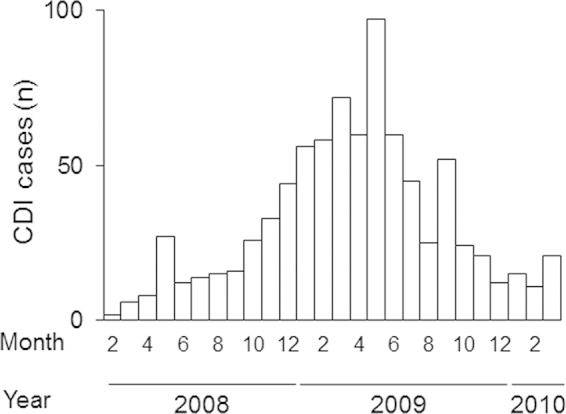
Epidemic curve for a CDI outbreak at a tertiary care hospital in Costa Rica, showing the numbers of CDI cases diagnosed (through clinical evidence and toxin detection) at San Juan de Dios Hospital during a 28-month period in 2008 to 2010.

**FIG 2:**
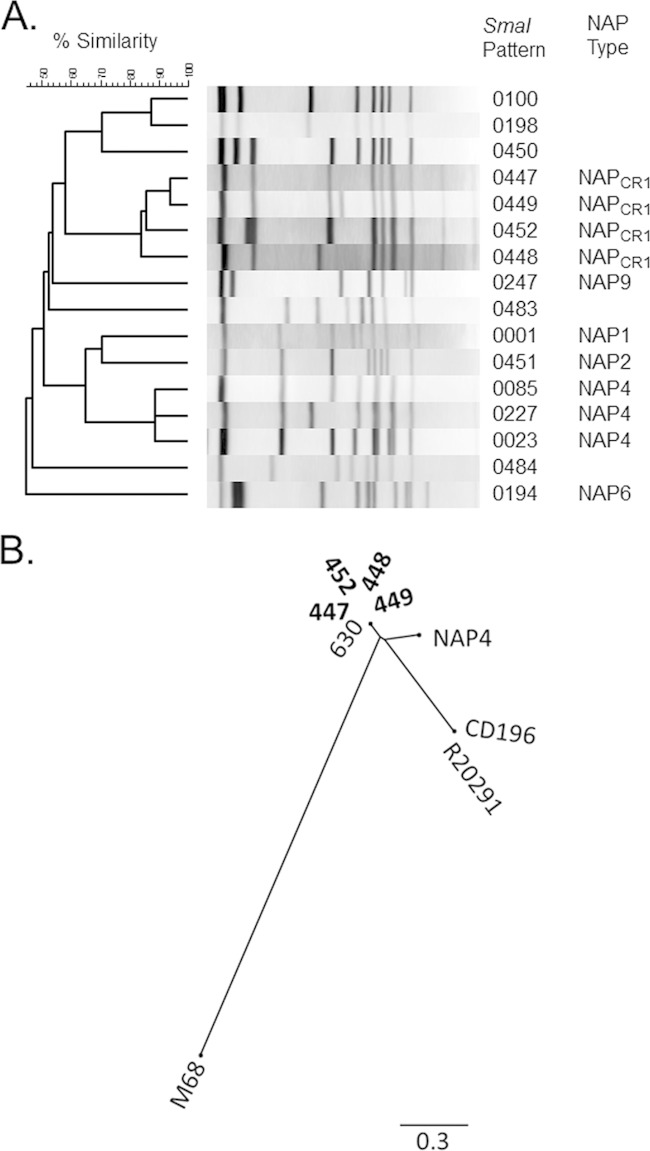
Molecular characterization of C. difficile isolates. (A) C. difficile strains (*n* = 57) isolated during the outbreak were typed by PFGE. Sixteen different SmaI macrorestriction patterns were detected and classified into the indicated NAP types. A previously undescribed NAP type was highly represented and was designated NAP_CR1_. (B) A phylogenomic tree based on core SNPs depicts the high level of genomic similarity of NAP_CR1_ strains (in bold) and their phylogenetic relationships to C. difficile 630, two NAP1 strains (R20291 and CD196), a NAP4 strain, and a NAP9 strain (M68). The scale distances correspond to the average number of substitutions per site.

During a C. difficile outbreak in a tertiary care hospital in Costa Rica (Fig. 1), 57 strains were isolated. These strains were assigned to 16 SmaI macrorestriction patterns using PFGE (Fig. 2A). Of those patterns, 7 belonged to previously described genotypes (NAP1, NAP2, NAP4, NAP6, and NAP9), whereas 9 did not match an existing NAP designation. Four of the unclassified SmaI PFGE patterns were frequently isolated and were preliminarily designated NAP_CR1_ (Fig. 2A). Future allocation of this genotype into the standard established NAP nomenclature requires the appearance of additional C. difficile strains displaying related SmaI macrorestriction patterns in other geographical locations. NAP1 and NAP_CR1_ strains accounted for the majority of isolates (45% and 31%, respectively). All of these strains were positive for *tcdA*, *tcdB*, and *tcdC*. In addition, NAP_CR1_ strains showed an 18-bp deletion in *tcdC*, as in NAP1 strains, but only NAP1 strains had a single-base-pair deletion at position 117.

### NAP_CR1_ and NAP1 strains are associated with increased disease severity.

To characterize the clinical and epidemiological spectra of CDIs produced by the different genotypes, patient data were classified into three groups, i.e., patients infected with NAP1 strains (*n* = 26), patients infected with NAP_CR1_ strains (*n* = 18), and patients infected with strains of other genotypes (*n* = 13). The average ages of patients were significantly different (Table 1); whereas NAP_CR1_ strains affected younger patients, NAP1 strains and strains of other genotypes affected older individuals. Patients infected with NAP_CR1_ strains presented higher white blood cell counts, and most of them were male. In general, there were no significant differences in the distributions of comorbidities. However, noticeable numbers of infections with NAP1 and NAP_CR1_ strains were associated with trauma (Table 2).

**TABLE 1:** Characteristics of patients with CDIs caused by different genotypes

Variable^*a*^	NAP1 (*n* = 26)	NAP_CR1_ (*n* = 18)	Other genotypes (*n* = 13)
Age (mean ± SD) (yr)	70 ± 22	48 ± 30^*b*^	60 ± 35
Hospital stay before onset of diarrhea (mean ± SD) (days)	11 ± 20	24 ± 47	12 ± 32

a SD, standard deviation.

b *P* < 0.05, compared to each other group, by one-way ANOVA with Bonferroni's correction.

**TABLE 2:** Univariate analysis of risk factors and outcomes of CDIs caused by different genotypes

Parameter	Proportion (%)			*P* (OR [95% CI])^*a*^		
	NAP1 (*n* = 26)	NAP_CR1_ (*n* = 18)	Other genotypes (*n* = 13)	NAP_CR1_ vs NAP1	NAP_CR1_ vs other genotypes	NAP1 vs other genotypes
Male	57.7	88.9	53.8	0.04^*b*^ (5.86 [1.11-0.95])	0.04^*b*^ (6.85 [1.10-42.75])	1.0 (0.85 [0.22-3.32])
Nosocomial CDI	96.1	100	69.2	1.0 (no OR^*c*^)	0.03^*b*^ (no OR^*c*^)	1 (1.11 [1.09-113.06])
Underlying disease						
Diabetes mellitus	23.1	16.7	7.7	0.71 (0.66 [0.14-3.11])	0.38 (3.6 [0.38-33.63])	0.38 (3.6 [0.38-33.63])
Cardiovascular disease	38.5	16.7	15.4	0.18 (0.32 [0.07-1.39])	0.66 (1.7 [0.25-11.58])	0.27 (3.43 [0.62-18.84])
Chronic obstructive pulmonary disease	11.5	11.1	15.4	1.0 (1 [0.15-6.97])	1.0 (1.0 [0.13-8.46])	1.0 (0.71 [0.10-4.93])
Malignancy	30.8	33.3	7.7	1.0 (0.88 [0.24-3.21])	0.04^*b*^ (9 [1.0-84.49])	0.22 (5.33 [0.59-48.30])
Trauma	3.8	27.8	23.1	0.03^*b*^ (9.61 [1.01-91.15])	1.0 (1.28 [0.25-6.69])	0.09 (0.13 [0.12-1.44])
Pharmacotherapy						
Antibiotic use within 8 wk prior to CDI	96.1	94.4	76.9	1.0 (0.68 [0.04-11.63])	0.28 (5.1 [0.46-55.89])	0.09 (7.5 [0.69-80.95])
Fluoroquinolone exposure within 8 wk prior to CDI	53.8	44.4	7.7	0.76 (1.25 [0.38-4.18])	0.04^*b*^ (9.6 [1.02-90.34])	0.006^*b*^ (14 [1.58-123.95])
Gastric acid suppressors	46.1	55.5	61.5	0.76 (0.68 [0.20-2.30])	1.0 (0.78 [0.18-3.34])	0.50 (0.54 [0.14-2.08])
Clinical features						
White blood cell count of >15,000 cells/μl	38.5	61.1	30.1	0.22 (2.5 [0.73-8.63])	0.15 (3.54 [0.78-16.03])	0.73 (1.41 [0.34-5.81])
Albumin level of <2.5 mg/dl	61.5	55.5	53.8	0.76 (0.78 [0.23-2.65])	0.72 (1.45 [0.35-6.11])	0.73 (1.38 [0.36-5.27])
Fever of >38°C	38.5	61.1	69.2	0.22 (2.5 [0.73-8.63])	0.71 (0.69 [0.15-3.16])	0.09 (0.28 [0.06-1.14])
Severe disease according to IDSA/SHEA criteria	50	55.5	15.4	0.76 (1.25 [0.37-4.17])	0.03^*b*^ (6.87 [1.17-40.38])	0.05^*b*^ (5.50 [1.01-29.85])
Severe disease according to criteria of Zar et al. (26)	69.2	72.2	30.7	0.55 (1.15 [0.31-4.35])	0.03^*b*^ (5.85 [1.22-27.99])	0.03^*b*^ (5.06 [1.20-21.42])
Recurrence	30.8	38.9	7.7	0.74 (1.43 [0.40-5.06])	0.04^*b*^ (9.6 [1.02-90.34])	0.03^*b*^ (8.8 [1.01-78.10])
30-day all-cause death	30.8	16.9	15.4	0.55 (0.32 [0.07-1.39])	0.84 (1.26 [0.8-4.33])	0.15 (2.5 [0.77-5.63])
30-day attributable death	26.9	16.9	0	0.49 (0.54 [0.12-2.46])	0.05^*b*^ (no OR^*c*^)	0.03^*b*^ (no OR^*c*^)

a *P* values were calculated using Fisher's exact test.

b Statistically significant (*P* ≤ 0.05).

c OR could not be calculated because one of the proportions was 0% or 100%.

CDIs with NAP_CR1_ (100%) and NAP1 (96%) strains were hospital acquired, whereas 31% of the cases caused by other genotypes were community-associated CDIs. Patients undergoing fluoroquinolone therapy were 10 or 14 times more likely to develop infections caused by NAP_CR1_ or NAP1 strains, respectively, than infections caused by other genotypes (NAP_CR1_ versus other genotypes, *P* = 0.04 [OR, 9.6]; NAP1 versus other genotypes, *P* = 0.006 [OR, 14]) (Table 2).

Infections with NAP_CR1_ and NAP1 strains were more likely to be associated with increased disease severity, according to the IDSA/SHEA criteria (NAP_CR1_ versus other genotypes, *P* = 0.03 [OR, 6.87 [95% CI, 1.17 to 40.38]]) and the criteria described by Zar et al. (26) (NAP_CR1_ versus other genotypes, *P* = 0.03 [OR, 5.85 [95% CI, 1.22 to 27.99]]) (Table 2). Recurrence was 10 and 9 times more likely in patients infected with NAP_CR1_ and NAP1 strains, respectively, than in patients infected with other genotypes (NAP_CR1_ versus other genotypes, *P* = 0.04 [OR, 9.6]; NAP1 versus other genotypes, *P* = 0.03 [OR, 8.8]) (Table 2). Similarly, the 30-day attributable mortality rates for NAP_CR1_ and NAP1 strains were significantly higher (17% and 27%, respectively) than that for strains of other genotypes (*P* = 0.05 and *P* = 0.03, respectively) (Table 2).

### Comparative genomic analysis of NAP_CR1_ strains.

To further study the NAP_CR1_ variant, we performed whole-genome sequencing and comparative genomic analysis. All of the NAP_CR1_ strains are very closely related, as indicated by the finding of only 101 core SNPs in the ∼4.5-Mb genomes (Fig. 2B). When we compared NAP_CR1_ genomes to reference genomes from common C. difficile strains, the NAP_CR1_ strains were not related to NAP1 lineages (68,413 core SNPs). Instead, average nucleotide identity (ANI) of 99% and 405 SNPs distinguished the core genomes of NAP_CR1_ and C. difficile strain 630. By ribotyping we determined that NAP_CR1_ belongs to ribotype 012, and by MLST we determined that this strain belongs to ST54 (data not shown). Strain 630 belongs to the same typing groups, which confirms the close relationship with NAP_CR1_.

NAP_CR1_ strains have more laterally acquired DNA than close relatives. NAP_CR1_ has about 6% more DNA and 10% more predicted proteins than C. difficile strain 630 (4,549,499 bp and 4,201 proteins versus 4,290,252 bp and 3,819 proteins). Compared to C. difficile strain 630, NAP_CR1_ has almost twice as many functions from the category of phages, prophages, transposable elements, and plasmids (see Table S1 in the supplemental material). Further functional differences were mostly related to DNA/RNA metabolism and regulation and cell signaling (see Table S1 in the supplemental material). Metabolic reconstruction of the NAP_CR1_ and C. difficile strain 630 genomes revealed that the NAP_CR1_ genotype has genes from 14 different categories not present in C. difficile strain 630. Six of these categories are associated with phages, and an additional one has to do with antibiotic resistance. In contrast, NAP_CR1_ lacks genes related to chorismate synthesis, Ton and Tol transport, phage DNA synthesis, and phage-packaging machinery. NAP_CR1_ and C. difficile strain 630 have 338 and 161 unique genes, respectively (see Tables S2 and S3 in the supplemental material). Almost all unique NAP_CR1_ sequences encode hypothetical proteins and cluster in contigs carrying phage genes or, to a minor extent, antibiotic resistance genes (see Table S2 in the supplemental material).

### NAP_CR1_ strains display a virulent phenotype.

**FIG 3:**
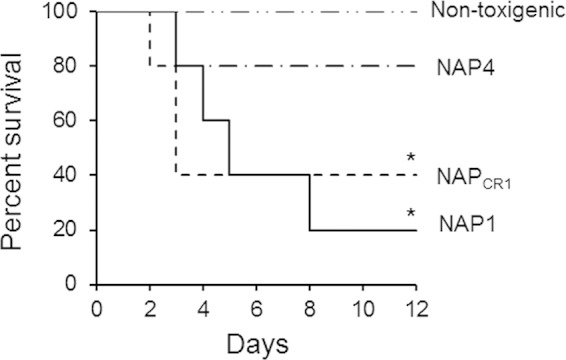
Kaplan-Meier survival curves for hamsters infected with different clinical genotypes of C. difficile. Groups of 5 Syrian Golden hamsters previously treated with clindamycin were orally inoculated with spores from the indicated genotypes. Hamsters were monitored at 12-h intervals for signs of C. difficile infection, and the numbers of dead animals were recorded. C. difficile isolates obtained from fecal pellets were typed by PFGE to confirm the inoculated strain. *, *P* < 0.05 (Mantel-Cox test).

The pathogenic potential of NAP_CR1_ strains was compared to that of other genotypes isolated in the outbreak by using two animal models, i.e., the hamster-spore infection model and murine ligated ileal loops inoculated with bacteria-free supernatant. In order to determine the rates of deaths induced by each genotype, clindamycin-treated hamsters were infected with spores. NAP_CR1_, NAP1, NAP4, and nontoxigenic strains colonized 100% of the hamsters within 6 days. The survival rates of hamsters inoculated with NAP_CR1_ and NAP1 spores declined rapidly, with the groups reaching 40% survival at day 5 and day 3, respectively (Fig. 3). In contrast, the survival rate for hamsters inoculated with NAP4 spores was 80% at 12 days after inoculation (Fig. 3). All animals inoculated with spores from nontoxigenic strains survived the duration of the experiments (Fig. 3). Inoculation of non-antibiotic-treated hamsters with spores from all of the genotype groups failed to result in colonization.

**FIG 4:**
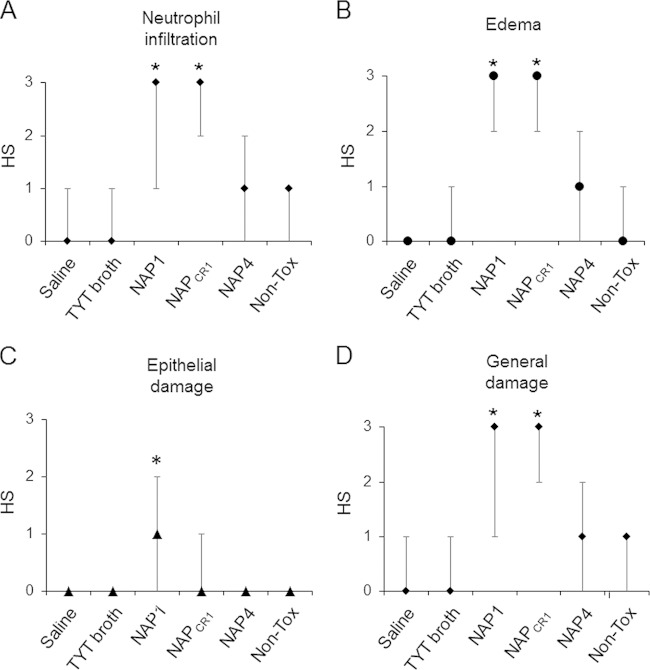
Quantification of histopathological effects of bacteria-free supernatants of the C. difficile genotypes in the murine ligated ileal loop model. Bacteria-free supernatants (48-h growth) of representative strains from the indicated genotypes were prepared in TYT broth. Six to 8 mice per group were inoculated with 0.3 ml of the indicated supernatant in ligated ileal loops. Four hours after inoculation, the mice were sacrificed and the severity of the histopathological alterations was scored on coded slides, using a histopathological score (HS) scale of 1 (mild) to 3 (severe) for neutrophil infiltration (A), edema (B), and epithelial damage (C); the general damage induced in the indicated groups was determined as the median of all scores (D). Non-Tox, nontoxigenic. *, *P* < 0.05, compared to the groups without asterisks (Kruskal-Wallis test and Dunn's multiple-comparison test).

In the ligated ileal loop model, the NAP1 supernatant induced strong inflammatory reactions, measured as the normalized weights of the ligated ileal loops (100 ± 15 mg/cm). The NAP_CR1_ supernatant induced a less severe reaction (66 ± 10 mg/cm) than that induced by the NAP1 strain; however, the response elicited was stronger than that induced by the NAP4 supernatant (54 ± 6 mg/cm). Histological analyses indicated that the NAP_CR1_ and NAP1 supernatants induced greater inflammatory cell infiltration and edema than did the NAP4 supernatants (Fig. 4). Only the NAP1 supernatant induced intense mucosal disruption with epithelial damage (Fig. 4).

We measured the concentration of myeloperoxidase (MPO) activity as an indicator of tissue neutrophil infiltration and the levels of IL-1β, IL-6, and TNF-α to indicate immune activation at the ileal tissue level. NAP_CR1_ and NAP1 supernatants caused statistically significant increases in MPO activity, in contrast to the NAP4 supernatant, which elicited a reaction similar to that observed with a nontoxigenic control (see Fig. S1 in the supplemental material). IL-6 and TNF-α levels were strongly induced in ileal tissue by NAP_CR1_ and NAP1 supernatants. Again, the NAP4 supernatant induced a reaction similar to that observed with the nontoxigenic control (see Fig. S1 in the supplemental material). IL-1β expression was highly induced by the NAP1 supernatant, compared to the other C. difficile strains (see Fig. S1 in the supplemental material).

### NAP_CR1_ strains are highly resistant to fluoroquinolones.

NAP_CR1_ and NAP1 strains were resistant to moxifloxacin and levofloxacin, whereas almost all of the other genotypes were susceptible to these antibiotics (see Fig. S2 in the supplemental material). In addition, the NAP_CR1_ but not NAP1 strains were also resistant to clindamycin and rifampin (see Fig. S2 in the supplemental material). Since fluoroquinolone resistance in C. difficile has been attributed to point mutations in either *gyrA* or *gyrB*, we sequenced those genes in selected strains from the outbreak (49). As reported previously, NAP1 strains presented the Thr82-to-Ile amino acid substitution in GyrA (50, 51). Among fluoroquinolone-susceptible control isolates (NAP4 and NAP6), no mutations were detected in either *gyrA* or *gyrB*.

### NAP_CR1_ strains do not produce increased amounts of toxins.

**FIG 5:**
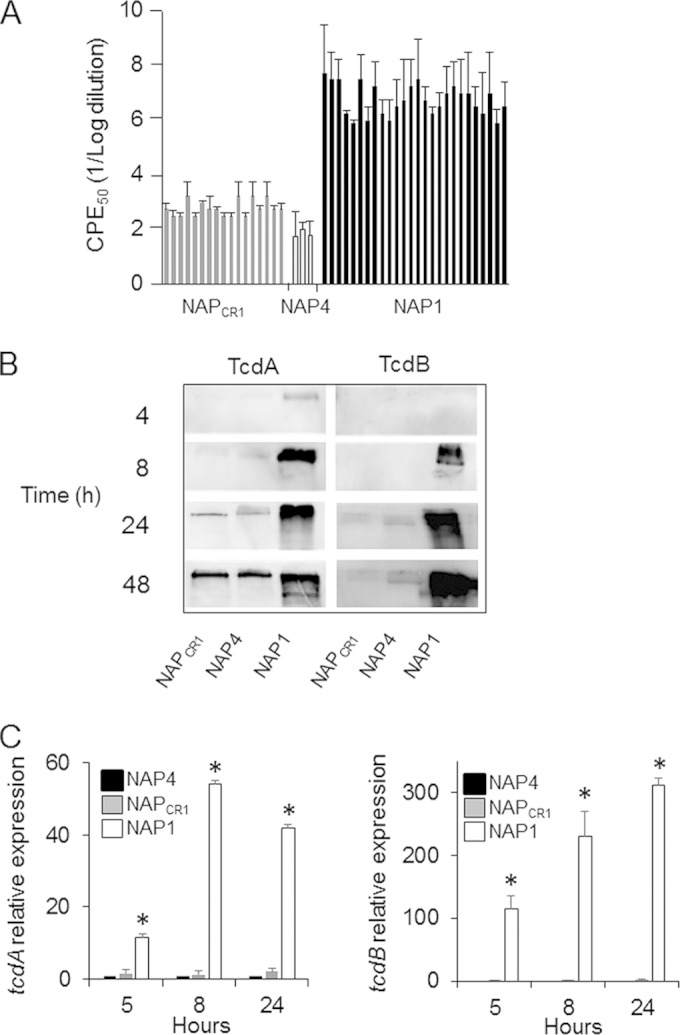
Quantification of toxin production by the different genotype groups. (A) Twenty-four-hour bacteria-free supernatants were titrated in 10-fold dilutions on HeLa cell monolayers. Twenty-four hours after inoculation with the indicated supernatant, the dilution inducing a cytopathic effect (CPE) for 50% of the cells was calculated by visual examination under a microscope. Each bar represents the CPE_50_ of one strain. (B) Proteins from bacteria-free supernatants obtained at the indicated times were precipitated and separated by 7.5% SDS-PAGE. Proteins were electrotransferred to PVDF membranes and probed with monoclonal antibodies against TcdA and TcdB. (C) Total RNA was prepared from the indicated strains at 5 and 8 h during the growth cycle. RNA was retrotranscribed, and cDNA was quantified by real-time PCR using primers specific for *tcdA* and *tcdB*. *, *P* < 0.05 (one-way analysis of variance [ANOVA] with Bonferroni's correction).

We compared the ability to produce and to secrete toxins in selected strains isolated during the outbreak by measuring toxin activity, TcdA and TcdB protein levels, and expression of *tcdA* and *tcdB* transcripts. NAP1 strains consistently gave higher cytotoxic titers than did NAP_CR1_ and NAP4 strains (Fig. 5A). Supernatants from each group of strains were collected at different times during the growth cycle, and the amounts of toxin were determined by Western blotting. TcdA was detected in NAP1 supernatants within the first 4 h, and concentrations increased steadily up to 24 h (Fig. 5B). The amounts of TcdA were lower in NAP_CR1_ and NAP4 supernatants at all times, being barely detectable at 8 h and increasing up to 48 h (Fig. 5B). TcdB was detected in NAP1 supernatants at 8 h, and its concentration peaked at 24 h. In contrast, TcdB was detectable in supernatants from NAP_CR1_ and NAP4 strains only at 24 h, at lower concentrations (Fig. 5B). The *tcdA* and *tcdB* mRNAs were quantified by real-time PCR, and the levels of both transcripts were higher in NAP1 strains than in NAP_CR1_ and NAP4 strains at all times tested (Fig. 5C).

## DISCUSSION

The incidence and severity of CDIs are increasing throughout the world (52, 53), a phenomenon that is partly due to the emergence of epidemic C. difficile strains (13, 14, 18, 54, 55). Here we described the emergence of a C. difficile strain with genetic, clinical, and virulence features that resemble those of NAP1 strains but within a C. difficile lineage, ribotype 12/ST54, for which no epidemic strains have been reported previously.

The NAP_CR1_ genotype displays a more aggressive phenotype both in clinically infected patients and in animal models. Patients whose CDIs were caused by NAP_CR1_ strains were younger than those affected by NAP1 and other genotypes and interestingly, as with the highly virulent NAP1 strains, a significant percentage of NAP_CR1_ cases were associated with trauma as a risk factor. These two epidemiological characteristics depart from the classic profile for patients affected by C. difficile, which includes age of >65 years and chronic debilitating diseases as risk factors. In addition, patients affected by NAP_CR1_ presented recurrence rates and 30-day attributable mortality rates as high as those presented by patients affected by NAP1. Furthermore, a majority of patients affected by NAP_CR1_ presented white blood cell counts higher than 15,000 cells/μl, supporting the proinflammatory nature of the response elicited by this particular strain (see below). Thus, the clinical picture induced by NAP_CR1_ strains, as measured using different standardized clinical criteria, is as severe as that induced by strains of the epidemic NAP1 genotype. The NAP_CR1_ strain was isolated from approximately one-third of the patients involved in the outbreak described in this report. The epidemic genotype, NAP1, was detected in a similar percentage, whereas other genotypes were less frequently represented. It is important to note that in this study we worked with toxin-positive samples; considering that NAP_CR1_ strains do not produce increased amounts of toxins, it is possible that some milder cases involving this group of strains were missed and that the overall prevalence of NAP_CR1_ is even higher than that reported here.

The more virulent phenotype of NAP_CR1_ was also demonstrated with animals challenged orally with spores; the NAP_CR1_ strain was as virulent as the NAP1 strain in terms of the ability to decrease the survival rate for clindamycin-treated hamsters. In contrast, in this model the NAP1 strain has consistently displayed increased lethality versus strain 630 (41, 56, 57). Considering the close phylogenetic relationship between NAP_CR1_ and strain 630 and the fact that both strains are resistant to clindamycin, the different behavior in the hamster model indicates that the latter has acquired virulence factors that increase its pathogenicity. These observations also indicate a positive correlation between severe CDI outcomes in humans and increased lethality of epidemic strains in animal models, thus demonstrating the increased virulence of NAP_CR1_.

A factor consistently associated with the selection and spread of NAP1 strains is resistance to fluoroquinolones (6, 49, 50), and it is well documented that restriction in the usage of these antibiotics results in decreases of CDIs (58, 59). In this work, we report that fluoroquinolone resistance is shared by NAP_CR1_ and NAP1 strains. The NAP_CR1_ strains harbor the same mutation in *gyrA* as reported for fluoroquinolone-resistant NAP1 strains, suggesting that the two genotypes share the same mechanism of resistance. This mutation has not been previously reported in ribotype 12/ST54 (60, 61). This observation reflects the successful nature of this mutation in conferring fluoroquinolone resistance to C. difficile in response to the selection pressure imposed by the use of this family of antibiotics. It is clear that, while fluoroquinolone resistance is not a molecular determinant of pathogenicity, the high level of antibiotic resistance introduces a major selection force that favors the dissemination of epidemic and endemic strains and thus is a procolonization factor (16).

The pathogenic phenotype of NAP1 strains has been attributed to increased production of TcdA and TcdB (9, 62). Some studies have suggested that this characteristic is related to deletions in the *tcdC* gene (8, 63). Thus, we hypothesized that the aggressive phenotype demonstrated by the NAP_CR1_ strain, which also presents an 18-bp deletion in *tcdC*, would depend on increased toxin production. This was clearly not the case, however, since the levels of TcdA and TcdB produced by the NAP_CR1_ strain were significantly lower than the levels of toxins produced by NAP1 strains. This lack of correlation between *tcdC* deletions and increased toxin production has been documented previously (11, 64). Despite the presence of the 18-bp deletion in *tcdC*, the NAP_CR1_ genotype does not exhibit the frameshift mutation at position 117, which probably has a greater impact on the functionality of TcdC as a negative regulator of TcdA and TcdB expression (63). Thus, molecular factors other than increased levels of toxins could account for the increased virulence observed for the NAP_CR1_ strain. These factors could involve metabolic and/or pathogenic adaptations that allow the bacteria to colonize the intestines of affected patients more efficiently. In the murine ligated loop model, we found that the inflammatory response elicited by NAP_CR1_ supernatants was almost as strong as that induced by NAP1 supernatants, despite the remarkable differences in TcdA and TcdB concentrations between the two strains. Thus, we present evidence that the emerging NAP_CR1_ genotype is able to induce inflammatory reactions (neutrophil recruitment and cytokine induction) and epithelial damage usually attributed to TcdA and TcdB. This capacity may be associated with other virulence factors that have not yet been described for NAP_CR1_ and that would be responsible for the aggressive pathological response. This hypothesis is in agreement with recent reports demonstrating several effects of C. difficile on host immunity that are toxin independent (65–69). Since the NAP_CR1_ strain belongs to the ribotype 012/ST54 group, for which no epidemic strains have been reported previously, its increased virulence could reside in the additional genomic content found in this genotype, in comparison with C. difficile strain 630. It is difficult at this point to assign the virulent phenotype to a particular set of genes, due to the large number and hypothetical nature of these additional open reading frames. However, the abundance and diversity of prophages found in the emerging NAP_CR1_ genotype could play a role in the increased virulence of this strain, since these genetic elements have been found previously to be involved in the regulation of virulence-associated genes (70–73). In this scenario, new virulent strains may arise through the acquisition of foreign DNA, with the ability to modulate immune responses, to tolerate antibiotics, and to regulate expression of virulence traits.

In conclusion, we described an emerging strain that possesses increased virulence potential due to the acquisition of laterally acquired genes and its ability to induce an exacerbated inflammatory response in the gastrointestinal mucosa, through currently unknown mechanisms. The emergence of strains with increased virulence is of importance in the surveillance of C. difficile outbreaks associated with both endemic and epidemic strains.

## Supplementary Material

Supplemental material
