# Supplementary material for: Emergence of an Outbreak-Associated Clostridium difficile Variant with Increased Virulence
Source: J Clin Microbiol. 2015 Mar 18;53(4):1216–26. doi: 10.1128/JCM.03058-14 (PMC4365207; doi:10.1128/JCM.03058-14)
Supplement: Supplemental material [file supp_53_4_1216__index.html]

Supplemental material 

# Emergence of an Outbreak-Associated Clostridium difficile Variant with Increased Virulence

## Supplemental material

**Files in this Data Supplement:**

- Supplemental file 1 -

  Fig. S1 (Effect of bacterial free supernatants on inflammatory cytokines and myeloperoxidase activity in the ligated murine ileal model) and S2 (Antimicrobial resistance profile of the *C. difficile* genotypes identified during the outbreak) and Tables S1 (Comparison of genomic features of a representative NAPCR1 strain and CD630), S2 (Genes in NAPCR that are not found in the *C. difficile* 630 strain by genomic comparison), and S3 (Genes in the *C. difficile* 630 strain that are not found in NAPCR1 by genomic comparison)

  PDF, 825K
