# Supplementary material for: Emergence of an Outbreak-Associated Clostridium difficile Variant with Increased Virulence
Source: J Clin Microbiol. 2015 Mar 18;53(4):1216–26. doi: 10.1128/JCM.03058-14 (PMC4365207; doi:10.1128/JCM.03058-14)
Supplement: Supplemental material [file JCM.03058-14_zjm999094154so1.pdf]

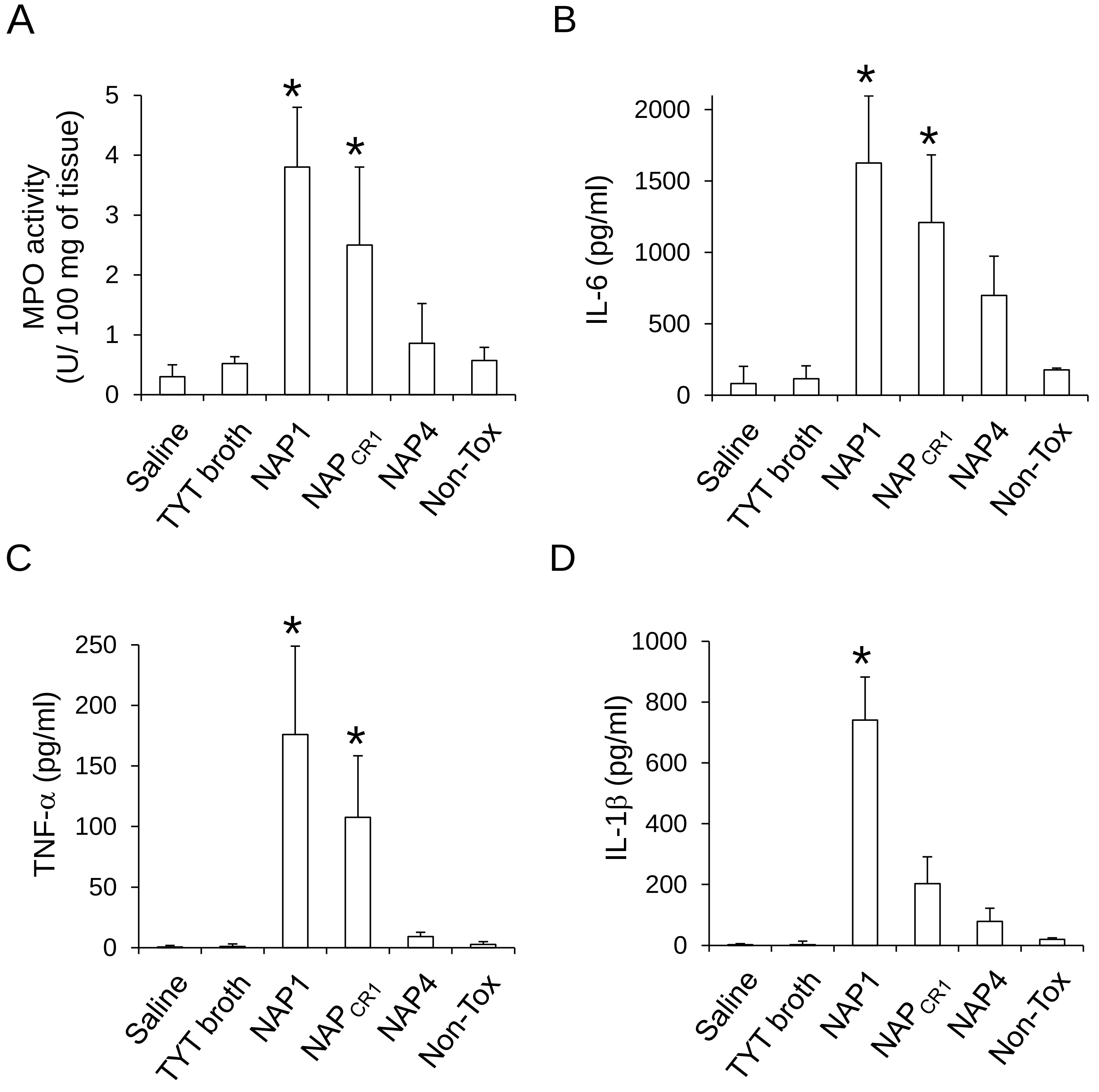

FIGURE S1. Effect of bacterial free supernatants on inflammatory cytokines and myeloperoxidase (MPO) activity in the ligated murine ileal model. Samples from ligated ileal groups prepared as indicated in figure 3 legend were processed for determination of MPO activity (A) or immunoenzymatic quantification of IL-6 (B), TNF-a (C) or IL-1b (D). **P* < 0,05, compared to the groups without asterisk (One-way ANOVA with Bonferroni´s correction).

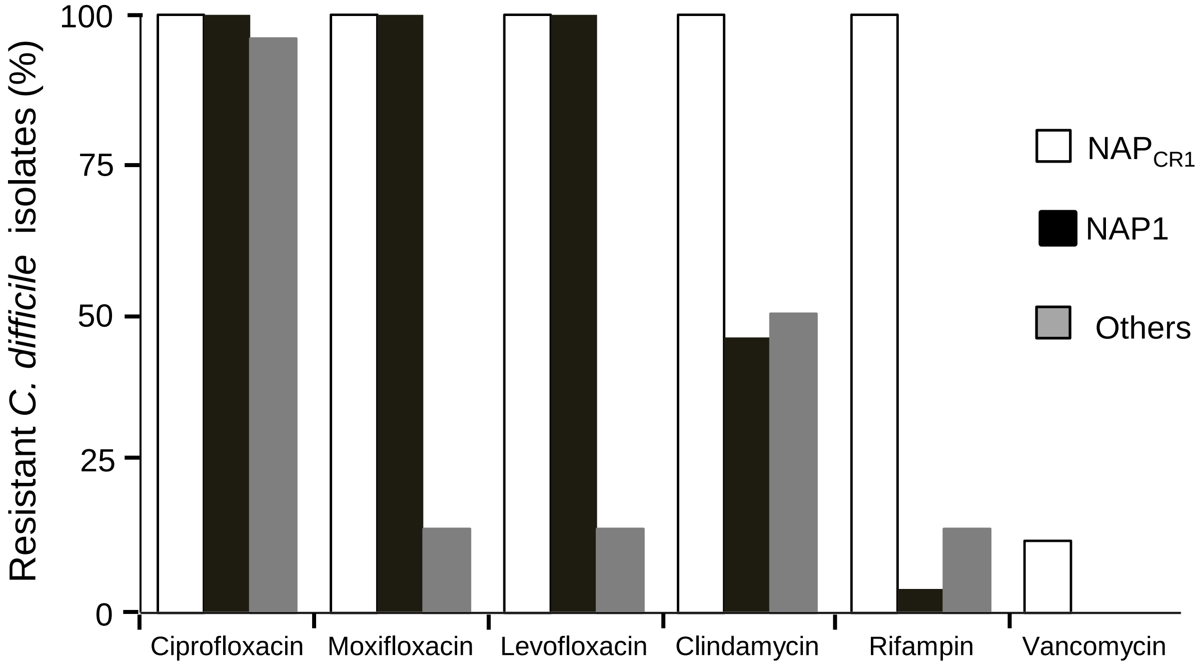

FIGURE S2 Antimicrobial resistance profile of the *C. difficile* genotypes identified during the outbreak. The 57 strains were divided in three groups: NAP_{CR1} (n=18), NAP1 (n=26) and other genotypes (n=13). Antibiotic susceptibility test was determined by agar dilution. The percentage of resistant strains in each group is indicated.

TABLE S1. Comparison of genomic features of a representative NAP_{CR1} strain and CD630. Counts indicate the number of genes assigned to each category of functional roles.

**Subsystem Feature***	**Counts**	
	**NAP_{CR1}**	**630**
Phages, Prophages, Transposable elements, Plasmids	**55**	34
DNA Metabolism	**130**	116
Cell Wall and Capsule	150	150
Virulence, Disease and Defense	100	100
Potassium metabolism	12	12
Miscellaneous	11	11
Cofactors, Vitamins, Prosthetic Groups, Pigments	252	249
Membrane Transport	54	68
Iron acquisition and metabolism	32	31
RNA Metabolism	105	102
Nucleosides and Nucleotides	150	146
Protein Metabolism	248	249
Cell Division and Cell Cycle	44	41
Motility and Chemotaxis	68	68
Regulation and Cell signaling	31	24
Fatty Acids, Lipids, and Isoprenoids	95	95
Nitrogen Metabolism	9	9
Dormancy and Sporulation	68	68
Respiration	80	80
Stress Response	64	68
Metabolism of Aromatic Compounds	11	11
Amino Acids and Derivatives	297	301
Sulfur Metabolism	24	24
Phosphorus Metabolism	45	43
Carbohydrates	422	427

*Details for subsystem and functional role assignment for the genes of each strain using SEED and RAST servers are listed in *Materials and Methods*.

TABLE S2. Genes in NAP_{CR} that are not found in *C. difficile* 630 strain by genomic comparison

Gene ID	Protein length	Contig	Function
fig|1496.572.peg.8	43	1	hypothetical protein
fig|1496.572.peg.38	38		hypothetical protein
fig|1496.572.peg.46	49		hypothetical protein
fig|1496.572.peg.73	54		FIG00516771: hypothetical protein
fig|1496.572.peg.139	41		hypothetical protein
fig|1496.572.peg.231	40		hypothetical protein
fig|1496.572.peg.242	83		hypothetical protein
fig|1496.572.peg.248	51		hypothetical protein
fig|1496.572.peg.294	50		hypothetical protein
fig|1496.572.peg.374	52		FIG00513157: hypothetical protein
fig|1496.572.peg.400	39		hypothetical protein
fig|1496.572.peg.441	39		hypothetical protein
fig|1496.572.peg.460	53		FIG00517932: hypothetical protein
fig|1496.572.peg.490	39		hypothetical protein
fig|1496.572.peg.629	105	2	FIG00528816: hypothetical protein
fig|1496.572.peg.635	138		Recombinase
fig|1496.572.peg.637	290		FIG00630629: hypothetical protein
fig|1496.572.peg.639	303		putative aminoglycoside 6-adenylyltansferase
fig|1496.572.peg.641	265		Aminoglycoside phosphotransferase
fig|1496.572.peg.643	220		5-amino-6-(5-phosphoribosylamino)uracil reductase (EC 1.1.1.193)
fig|1496.572.peg.645	124		hypothetical protein
fig|1496.572.peg.646	150		hypothetical protein
fig|1496.572.peg.647	48		hypothetical protein
fig|1496.572.peg.648	89		Iron-dependent repressor IdeR/DtxR
fig|1496.572.peg.651	49		hypothetical protein

fig|1496.572.peg.652	99	hypothetical protein
fig|1496.572.peg.653	229	hypothetical protein
fig|1496.572.peg.656	238	hypothetical protein
fig|1496.572.peg.658	60	hypothetical protein
fig|1496.572.peg.659	200	hypothetical protein
fig|1496.572.peg.661	72	hypothetical protein
fig|1496.572.peg.663	60	hypothetical protein
fig|1496.572.peg.664	64	hypothetical protein
fig|1496.572.peg.670	41	hypothetical protein
fig|1496.572.peg.672	63	FIG00514266: hypothetical protein
fig|1496.572.peg.677	99	FIG00521858: hypothetical protein
fig|1496.572.peg.678	204	hypothetical protein
fig|1496.572.peg.679	162	ADP-heptose synthase (EC 2.7.-.-) / D-glycero-beta-D-manno-heptose 7-phosphate kinase
fig|1496.572.peg.687	53	hypothetical protein
fig|1496.572.peg.688	110	hypothetical protein
fig|1496.572.peg.689	200	Chromosome (plasmid) partitioning protein ParB
fig|1496.572.peg.695	99	hypothetical protein
fig|1496.572.peg.523	78	hypothetical protein
fig|1496.572.peg.525	109	hypothetical protein
fig|1496.572.peg.581	40	hypothetical protein
fig|1496.572.peg.592	45	hypothetical protein
fig|1496.572.peg.596	66	hypothetical protein
fig|1496.572.peg.597	57	hypothetical protein
fig|1496.572.peg.599	46	hypothetical protein
fig|1496.572.peg.600	56	Transcriptional regulator
fig|1496.572.peg.601	59	Phage protein
fig|1496.572.peg.606	40	hypothetical protein
fig|1496.572.peg.607	82	hypothetical protein
fig|1496.572.peg.618	53	hypothetical protein

fig|1496.572.peg.620	42		hypothetical protein
fig|1496.572.peg.624	80		hypothetical protein
fig|1496.572.peg.627	38		hypothetical protein
fig|1496.572.peg.764	48	3	hypothetical protein
fig|1496.572.peg.798	48		hypothetical protein
fig|1496.572.peg.822	65		hypothetical protein
fig|1496.572.peg.922	48	4	FIG00520514: hypothetical protein
fig|1496.572.peg.959	43		hypothetical protein
fig|1496.572.peg.1021	38	5	hypothetical protein
fig|1496.572.peg.1059	52		FIG00527335: hypothetical protein
fig|1496.572.peg.1063	43		hypothetical protein
fig|1496.572.peg.1190	38	6	hypothetical protein
fig|1496.572.peg.1242	40		hypothetical protein
fig|1496.572.peg.1266	40	7	hypothetical protein
fig|1496.572.peg.1270	237		hypothetical protein
fig|1496.572.peg.1271	387		hypothetical protein
fig|1496.572.peg.1275	53		hypothetical protein
fig|1496.572.peg.1276	96		hypothetical protein
fig|1496.572.peg.1277	120		hypothetical protein
fig|1496.572.peg.1282	146		hypothetical protein
fig|1496.572.peg.1283	150		hypothetical protein
fig|1496.572.peg.1284	268		Phage protein
fig|1496.572.peg.1285	273		Phage protein
fig|1496.572.peg.1286	268		hypothetical protein

fig|1496.572.peg.1287	165	hypothetical protein
fig|1496.572.peg.1288	331	hypothetical protein
fig|1496.572.peg.1289	212	hypothetical protein
fig|1496.572.peg.1290	198	hypothetical protein
fig|1496.572.peg.1291	324	hypothetical protein
fig|1496.572.peg.1292	163	hypothetical protein
fig|1496.572.peg.1294	76	hypothetical protein
fig|1496.572.peg.1295	495	hypothetical protein
fig|1496.572.peg.1296	591	unknown
fig|1496.572.peg.1297	287	unknown
fig|1496.572.peg.1298	398	Phage protein
fig|1496.572.peg.1299	63	hypothetical protein
fig|1496.572.peg.1300	206	hypothetical protein
fig|1496.572.peg.1301	185	hypothetical protein
fig|1496.572.peg.1302	211	hypothetical protein
fig|1496.572.peg.1303	102	hypothetical protein
fig|1496.572.peg.1304	401	hypothetical protein
fig|1496.572.peg.1305	65	hypothetical protein
fig|1496.572.peg.1306	80	hypothetical protein
fig|1496.572.peg.1307	209	hypothetical protein
fig|1496.572.peg.1308	315	hypothetical protein
fig|1496.572.peg.1309	46	hypothetical protein
fig|1496.572.peg.1313	63	hypothetical protein
fig|1496.572.peg.1314	86	hypothetical protein
fig|1496.572.peg.1315	47	hypothetical protein
fig|1496.572.peg.1316	115	hypothetical protein
fig|1496.572.peg.1317	79	hypothetical protein
fig|1496.572.peg.1319	113	hypothetical protein
fig|1496.572.peg.1320	195	hypothetical protein

fig|1496.572.peg.1321	162	hypothetical protein
fig|1496.572.peg.1322	39	hypothetical protein
fig|1496.572.peg.1323	361	hypothetical protein
fig|1496.572.peg.1324	169	hypothetical protein
fig|1496.572.peg.1325	101	hypothetical protein
fig|1496.572.peg.1326	111	hypothetical protein
fig|1496.572.peg.1327	178	hypothetical protein
fig|1496.572.peg.1328	175	hypothetical protein
fig|1496.572.peg.1331	58	hypothetical protein
fig|1496.572.peg.1332	123	Death on curing protein, Doc toxin
fig|1496.572.peg.1334	298	hypothetical protein
fig|1496.572.peg.1335	113	hypothetical protein
fig|1496.572.peg.1336	47	hypothetical protein
fig|1496.572.peg.1337	78	hypothetical protein
fig|1496.572.peg.1338	138	hypothetical protein
fig|1496.572.peg.1339	65	hypothetical protein
fig|1496.572.peg.1340	39	hypothetical protein
fig|1496.572.peg.1341	181	RNA:NAD 2'-phosphotransferase
fig|1496.572.peg.1342	109	hypothetical protein
fig|1496.572.peg.1344	49	hypothetical protein
fig|1496.572.peg.1345	62	hypothetical protein
fig|1496.572.peg.1346	428	DNA ligase (ATP) (EC 6.5.1.1)
fig|1496.572.peg.1347	67	hypothetical protein
fig|1496.572.peg.1348	196	hypothetical protein
fig|1496.572.peg.1349	63	hypothetical protein
fig|1496.572.peg.1350	337	hypothetical protein
fig|1496.572.peg.1351	132	hypothetical protein
fig|1496.572.peg.1352	179	hypothetical protein
fig|1496.572.peg.1353	123	hypothetical protein

fig|1496.572.peg.1354	95	hypothetical protein
fig|1496.572.peg.1355	115	hypothetical protein
fig|1496.572.peg.1356	41	hypothetical protein
fig|1496.572.peg.1357	156	hypothetical protein
fig|1496.572.peg.1358	127	hypothetical protein
fig|1496.572.peg.1359	149	hypothetical protein
fig|1496.572.peg.1360	55	hypothetical protein
fig|1496.572.peg.1361	117	hypothetical protein
fig|1496.572.peg.1362	156	hypothetical protein
fig|1496.572.peg.1363	120	hypothetical protein
fig|1496.572.peg.1365	60	hypothetical protein
fig|1496.572.peg.1366	51	hypothetical protein
fig|1496.572.peg.1367	121	hypothetical protein
fig|1496.572.peg.1368	77	hypothetical protein
fig|1496.572.peg.1369	59	hypothetical protein
fig|1496.572.peg.1370	70	hypothetical protein
fig|1496.572.peg.1371	61	hypothetical protein
fig|1496.572.peg.1372	117	hypothetical protein
fig|1496.572.peg.1373	106	hypothetical protein
fig|1496.572.peg.1374	95	hypothetical protein
fig|1496.572.peg.1375	56	hypothetical protein
fig|1496.572.peg.1376	45	hypothetical protein
fig|1496.572.peg.1377	109	hypothetical protein
fig|1496.572.peg.1378	201	COG0030: Dimethyladenosine transferase (rRNA methylation)
fig|1496.572.peg.1379	70	hypothetical protein
fig|1496.572.peg.1380	84	hypothetical protein
fig|1496.572.peg.1382	374	Phage protein
fig|1496.572.peg.1383	144	hypothetical protein
fig|1496.572.peg.1384	52	hypothetical protein

fig|1496.572.peg.1386	222	hypothetical protein
fig|1496.572.peg.1387	57	hypothetical protein
fig|1496.572.peg.1388	282	hypothetical protein
fig|1496.572.peg.1389	192	Phage-associated recombinase
fig|1496.572.peg.1390	186	hypothetical protein
fig|1496.572.peg.1391	531	hypothetical protein
fig|1496.572.peg.1392	38	hypothetical protein
fig|1496.572.peg.1393	259	Phage toprim domain containing protein, YorJ B.subtilis homolog
fig|1496.572.peg.1396	101	hypothetical protein
fig|1496.572.peg.1397	380	hypothetical protein
fig|1496.572.peg.1399	125	hypothetical protein
fig|1496.572.peg.1400	44	hypothetical protein
fig|1496.572.peg.1401	82	hypothetical protein
fig|1496.572.peg.1402	178	hypothetical protein
fig|1496.572.peg.1403	269	hypothetical protein
fig|1496.572.peg.1404	51	hypothetical protein
fig|1496.572.peg.1405	65	hypothetical protein
fig|1496.572.peg.1406	209	serine/threonine protein phosphatase
fig|1496.572.peg.1407	85	hypothetical protein
fig|1496.572.peg.1408	95	hypothetical protein
fig|1496.572.peg.1409	270	Adenine-specific methyltransferase (EC 2.1.1.72)
fig|1496.572.peg.1410	121	hypothetical protein
fig|1496.572.peg.1411	191	Guanylate kinase (EC 2.7.4.8)
fig|1496.572.peg.1412	165	Crossover junction endodeoxyribonuclease RuvC (EC 3.1.22.4)
fig|1496.572.peg.1414	40	hypothetical protein
fig|1496.572.peg.1415	437	Il-IS_2, transposase # fragment
fig|1496.572.peg.1416	281	hypothetical protein
fig|1496.572.peg.1418	615	hypothetical protein
fig|1496.572.peg.1419	44	hypothetical protein

fig|1496.572.peg.1420	74		hypothetical protein
fig|1496.572.peg.1425	104		hypothetical protein
fig|1496.572.peg.1436	52		hypothetical protein
fig|1496.572.peg.1437	643		hypothetical protein
fig|1496.572.peg.1439	52		hypothetical protein
fig|1496.572.peg.1444	52	8	FIG00519437: hypothetical protein
fig|1496.572.peg.1447	40		hypothetical protein
fig|1496.572.peg.1465	50		FIG00520773: hypothetical protein
fig|1496.572.peg.1497	40		hypothetical protein
fig|1496.572.peg.1658	43	10	hypothetical protein
fig|1496.572.peg.1682	39		hypothetical protein
fig|1496.572.peg.1690	45		hypothetical protein
fig|1496.572.peg.1692	45		hypothetical protein
fig|1496.572.peg.1754	93	11	hypothetical protein
fig|1496.572.peg.1776	40		hypothetical protein
fig|1496.572.peg.1786	43		hypothetical protein
fig|1496.572.peg.1787	40		hypothetical protein
fig|1496.572.peg.1795	40		hypothetical protein
fig|1496.572.peg.1799	44		hypothetical protein
fig|1496.572.peg.1800	154		FIG00513238: hypothetical protein
fig|1496.572.peg.1825	45	12	hypothetical protein
fig|1496.572.peg.1856	48		hypothetical protein
fig|1496.572.peg.1868	43		hypothetical protein
fig|1496.572.peg.1903	49		hypothetical protein
fig|1496.572.peg.1918	41		hypothetical protein

fig|1496.572.peg.1933	38		hypothetical protein
fig|1496.572.peg.1935	54		FIG00520743: hypothetical protein
fig|1496.572.peg.1953	46		hypothetical protein
fig|1496.572.peg.1966	43		hypothetical protein
fig|1496.572.peg.2062	65		hypothetical protein
fig|1496.572.peg.2067	134		hypothetical protein
fig|1496.572.peg.2083	42		hypothetical protein
fig|1496.572.peg.2098	48		hypothetical protein
fig|1496.572.peg.2100	57		hypothetical protein
fig|1496.572.peg.2112	51		hypothetical protein
fig|1496.572.peg.2213	50	13	hypothetical protein
fig|1496.572.peg.2252	39	14	hypothetical protein
fig|1496.572.peg.2259	188		FIG00517580: hypothetical protein
fig|1496.572.peg.2285	53	15	Transcriptional regulator
fig|1496.572.peg.2286	57		Phage protein
fig|1496.572.peg.2287	48		hypothetical protein
fig|1496.572.peg.2289	39		hypothetical protein
fig|1496.572.peg.2290	145		Phage protein
fig|1496.572.peg.2291	85		hypothetical protein
fig|1496.572.peg.2292	251		Phage antirepressor protein
fig|1496.572.peg.2295	46		hypothetical protein
fig|1496.572.peg.2296	74		Phage protein
fig|1496.572.peg.2297	69		Phage protein
fig|1496.572.peg.2393	40	16	hypothetical protein

fig|1496.572.peg.2452	51	18	thioredoxin family protein
fig|1496.572.peg.2459	38		hypothetical protein
fig|1496.572.peg.2472	39		hypothetical protein
fig|1496.572.peg.2481	105		hypothetical protein
fig|1496.572.peg.2483	95		hypothetical protein
fig|1496.572.peg.2499	52	19	FIG00522519: hypothetical protein
fig|1496.572.peg.2530	50		hypothetical protein
fig|1496.572.peg.2534	38	20	hypothetical protein
fig|1496.572.peg.2561	134		Phage-related protein
fig|1496.572.peg.2562	46		hypothetical protein
fig|1496.572.peg.2564	70		hypothetical protein
fig|1496.572.peg.2565	58		Phage protein
fig|1496.572.peg.2566	57		Phage protein
fig|1496.572.peg.2567	263		Phage antirepressor protein
fig|1496.572.peg.2568	65		Phage protein
fig|1496.572.peg.2569	66		hypothetical protein
fig|1496.572.peg.2570	67		hypothetical protein
fig|1496.572.peg.2571	66		hypothetical protein
fig|1496.572.peg.2577	70	21	hypothetical protein
fig|1496.572.peg.2578	150		hypothetical protein
fig|1496.572.peg.2579	212		Lmo2276 protein
fig|1496.572.peg.2580	105		hypothetical protein
fig|1496.572.peg.2581	51		hypothetical protein
fig|1496.572.peg.2582	47		hypothetical protein
fig|1496.572.peg.2584	111		hypothetical protein
fig|1496.572.peg.2588	113		hypothetical protein

fig|1496.572.peg.2589	125		Transcriptional regulator, Cro/CI family
fig|1496.572.peg.2590	61		hypothetical protein
fig|1496.572.peg.2591	142		hypothetical protein
fig|1496.572.peg.2592	54		hypothetical protein
fig|1496.572.peg.2593	115		hypothetical protein
fig|1496.572.peg.2595	49		hypothetical protein
fig|1496.572.peg.2596	90		hypothetical protein
fig|1496.572.peg.2598	154		hypothetical protein
fig|1496.572.peg.2603	130		FIG00516442: hypothetical protein
fig|1496.572.peg.2604	550		Phage terminase, large subunit
fig|1496.572.peg.2605	385		Portal protein, phage associated
fig|1496.572.peg.2608	96		Phage protein
fig|1496.572.peg.2609	113		hypothetical protein
fig|1496.572.peg.2610	138		hypothetical protein
fig|1496.572.peg.2611	139		conserved protein
fig|1496.572.peg.2614	128		FIG00521161: hypothetical protein
fig|1496.572.peg.2615	56		hypothetical protein
fig|1496.572.peg.2625	508	22	Phage protein
fig|1496.572.peg.2626	540		Exonuclease SbcC
fig|1496.572.peg.2627	51		hypothetical protein
fig|1496.572.peg.2629	119		hypothetical protein
fig|1496.572.peg.2630	343		Phage major capsid protein
fig|1496.572.peg.2631	69		hypothetical protein
fig|1496.572.peg.2632	106		hypothetical protein
fig|1496.572.peg.2633	128		hypothetical protein
fig|1496.572.peg.2634	137		hypothetical protein
fig|1496.572.peg.2635	275		FIG00513450: hypothetical protein
fig|1496.572.peg.2636	68		hypothetical protein

fig|1496.572.peg.2638	138		Phage-like element PBSX protein xkdM
fig|1496.572.peg.2640	51		hypothetical protein
fig|1496.572.peg.2641	348		Phage tail length tape-measure protein
fig|1496.572.peg.2642	95		hypothetical protein
fig|1496.572.peg.2643	1049		Phage tail length tape-measure protein
fig|1496.572.peg.2646	321		Phage-like element PBSX protein xkdQ
fig|1496.572.peg.2647	122		hypothetical protein
fig|1496.572.peg.2654	57		hypothetical protein
fig|1496.572.peg.2655	189		FIG00513626: hypothetical protein
fig|1496.572.peg.2656	97		FIG00515668: hypothetical protein
fig|1496.572.peg.2733	40	23	hypothetical protein
fig|1496.572.peg.2743	38		hypothetical protein
fig|1496.572.peg.2838	56		FIG00514757: hypothetical protein
fig|1496.572.peg.2872	43		hypothetical protein
fig|1496.572.peg.2881	42		hypothetical protein
fig|1496.572.peg.2886	77		hypothetical protein
fig|1496.572.peg.2894	166		hypothetical protein
fig|1496.572.peg.2916	50		hypothetical protein
fig|1496.572.peg.2923	258	24	conserved domain protein
fig|1496.572.peg.2924	40		hypothetical protein
fig|1496.572.peg.2933	340		Phage major capsid protein #Fam0021
fig|1496.572.peg.2934	127		hypothetical protein
fig|1496.572.peg.2936	77		hypothetical protein
fig|1496.572.peg.2937	91		hypothetical protein
fig|1496.572.peg.2938	163		Phage protein
fig|1496.572.peg.2946	169		hypothetical protein
fig|1496.572.peg.2949	40		Phage protein

fig|1496.572.peg.2951	118		hypothetical protein
fig|1496.572.peg.2955	232		Phage protein
fig|1496.572.peg.2982	63	26	Phage protein
fig|1496.572.peg.2984	308		Phage protein
fig|1496.572.peg.2985	195		Phage protein
fig|1496.572.peg.2986	151		Phage protein
fig|1496.572.peg.2987	799		Phage terminase, large subunit
fig|1496.572.peg.2990	210		Phage protein
fig|1496.572.peg.2991	65		Phage protein
fig|1496.572.peg.2992	77		hypothetical protein
fig|1496.572.peg.2995	74		hypothetical protein
fig|1496.572.peg.2996	117		Phage protein
fig|1496.572.peg.2997	117		Phage protein
fig|1496.572.peg.3006	50		hypothetical protein
fig|1496.572.peg.3007	64		hypothetical protein
fig|1496.572.peg.3009	91		Phage protein
fig|1496.572.peg.3010	81		Phage protein
fig|1496.572.peg.3049	40	27	hypothetical protein
fig|1496.572.peg.3064	174	29	hypothetical protein
fig|1496.572.peg.3091	264	31	FIG00513202: hypothetical protein
fig|1496.572.peg.3092	40		hypothetical protein
fig|1496.572.peg.3094	86		Phage protein
fig|1496.572.peg.3097	245	32	hypothetical protein
fig|1496.572.peg.3098	155		hypothetical protein

fig|1496.572.peg.3099	164		hypothetical protein
fig|1496.572.peg.3101	76		FIG00632701: hypothetical protein
fig|1496.572.peg.3106	39	33	hypothetical protein
fig|1496.572.peg.3110	47	34	hypothetical protein
fig|1496.572.peg.3181	47		hypothetical protein
fig|1496.572.peg.3185	52		FIG00522352: hypothetical protein
fig|1496.572.peg.3188	46		hypothetical protein
fig|1496.572.peg.3203	422		Plasmid recombination, MobE mobilization protein
fig|1496.572.peg.3204	45		hypothetical protein
fig|1496.572.peg.3206	208		Chloramphenicol acetyltransferase (EC 2.3.1.28)
fig|1496.572.peg.3207	69		TnpV
fig|1496.572.peg.3242	46		hypothetical protein
fig|1496.572.peg.3257	188		hypothetical protein
fig|1496.572.peg.3272	105		FIG00528816: hypothetical protein
fig|1496.572.peg.3310	40		hypothetical protein
fig|1496.572.peg.3314	51		hypothetical protein
fig|1496.572.peg.3329	39	41	hypothetical protein
fig|1496.572.peg.3332	40		hypothetical protein
fig|1496.572.peg.3361	49		hypothetical protein
fig|1496.572.peg.3432	56		Dihydrofolate reductase (EC 1.5.1.3)
fig|1496.572.peg.3447	42		FIG00516082: hypothetical protein
fig|1496.572.peg.3469	49		hypothetical protein
fig|1496.572.peg.3506	110	42	Phage protein
fig|1496.572.peg.3512	63	44	Phage protein

fig|1496.572.peg.3534	45		hypothetical protein
fig|1496.572.peg.3622	45		hypothetical protein
fig|1496.572.peg.3677	38		hypothetical protein
fig|1496.572.peg.3689	391		Mobile element protein
fig|1496.572.peg.3691	480		6'-aminoglycoside N-acetyltransferase (EC 2.3.1.-) (AAC(6')) / 2"-aminoglycoside phosphotransferase (EC
fig|1496.572.peg.3711	51	45	hypothetical protein
fig|1496.572.peg.3773	51		hypothetical protein
fig|1496.572.peg.3855	74		FIG00513646: hypothetical protein
fig|1496.572.peg.4017	174	46	FIG01032097: hypothetical protein
fig|1496.572.peg.4027	47		hypothetical protein
fig|1496.572.peg.4054	51		hypothetical protein
fig|1496.572.peg.4063	64		FIG00517121: hypothetical protein
fig|1496.572.peg.4097	280	47	Diadenylate cyclase spyDAC; Bacterial checkpoint controller DisA with nucleotide-binding domain
fig|1496.572.peg.4129	42		hypothetical protein
fig|1496.572.peg.4142	86		4-hydroxyphenylacetate decarboxylase, small subunit (EC 4.1.1.83)
fig|1496.572.peg.4189	42		hypothetical protein

TABLE S3. Genes in *C. difficile* 630 strain that are not found in NAP_{CR1} by genomic comparison

Gene ID	Protein Length	Function
fig|1496.1.peg.15	98	
fig|1496.1.peg.41	80	
fig|1496.1.peg.72	54	
fig|1496.1.peg.90	59	
fig|1496.1.peg.128	68	
fig|1496.1.peg.131	75	
fig|1496.1.peg.145	71	
fig|1496.1.peg.153	69	
fig|1496.1.peg.158	34	
fig|1496.1.peg.161	77	
fig|1496.1.peg.298	59	
fig|1496.1.peg.342	76	
fig|1496.1.peg.357	62	
fig|1496.1.peg.378	69	
fig|1496.1.peg.400	81	
fig|1496.1.peg.403	46	
fig|1496.1.peg.416	66	
fig|1496.1.peg.452	77	
fig|1496.1.peg.498	70	
fig|1496.1.peg.514	190	
fig|1496.1.peg.515	187	hypothetical protein
fig|1496.1.peg.516	61	
fig|1496.1.peg.517	64	
fig|1496.1.peg.526	55	
fig|1496.1.peg.529	77	

fig|1496.1.peg.534	76	
fig|1496.1.peg.540	252	
fig|1496.1.peg.541	81	
fig|1496.1.peg.547	110	hypothetical protein
fig|1496.1.peg.581	67	
fig|1496.1.peg.585	76	
fig|1496.1.peg.593	79	
fig|1496.1.peg.594	64	
fig|1496.1.peg.663	73	
fig|1496.1.peg.668	59	
fig|1496.1.peg.711	77	
fig|1496.1.peg.736	59	
fig|1496.1.peg.816	70	
fig|1496.1.peg.834	78	
fig|1496.1.peg.836	104	
fig|1496.1.peg.862	59	
fig|1496.1.peg.865	52	
fig|1496.1.peg.880	59	
fig|1496.1.peg.932	61	
fig|1496.1.peg.960	43	
fig|1496.1.peg.965	99	
fig|1496.1.peg.980	95	hypothetical protein
fig|1496.1.peg.1010	68	
fig|1496.1.peg.1014	125	
fig|1496.1.peg.1017	120	
fig|1496.1.peg.1025	64	
fig|1496.1.peg.1046	58	
fig|1496.1.peg.1092	73	
fig|1496.1.peg.1144	61	

fig|1496.1.peg.1159	71	
fig|1496.1.peg.1199	99	
fig|1496.1.peg.1261	63	
fig|1496.1.peg.1271	66	
fig|1496.1.peg.1324	70	
fig|1496.1.peg.1345	61	
fig|1496.1.peg.1366	26	
fig|1496.1.peg.1375	63	
fig|1496.1.peg.1386	67	
fig|1496.1.peg.1422	58	
fig|1496.1.peg.1481	92	
fig|1496.1.peg.1508	84	
fig|1496.1.peg.3944	26	Peptide chain release factor 2 unshifted fragment
fig|1496.1.peg.1545	61	
fig|1496.1.peg.1559	63	
fig|1496.1.peg.1590	64	
fig|1496.1.peg.1655	59	
fig|1496.1.peg.1694	88	
fig|1496.1.peg.1700	62	
fig|1496.1.peg.1707	64	
fig|1496.1.peg.1713	99	
fig|1496.1.peg.1714	68	
fig|1496.1.peg.1715	93	
fig|1496.1.peg.1716	64	
fig|1496.1.peg.1727	77	
fig|1496.1.peg.1788	46	
fig|1496.1.peg.1795	73	
fig|1496.1.peg.1836	68	
fig|1496.1.peg.1839	79	

fig|1496.1.peg.1891	74	
fig|1496.1.peg.1896	66	
fig|1496.1.peg.1910	85	
fig|1496.1.peg.1957	63	
fig|1496.1.peg.1960	70	
fig|1496.1.peg.1969	130	
fig|1496.1.peg.1980	72	
fig|1496.1.peg.1982	76	
fig|1496.1.peg.2002	63	
fig|1496.1.peg.2006	61	
fig|1496.1.peg.2027	86	threonine dehydratase biosynthetic
fig|1496.1.peg.2040	93	
fig|1496.1.peg.2054	77	
fig|1496.1.peg.2060	117	
fig|1496.1.peg.2061	63	
fig|1496.1.peg.2067	78	
fig|1496.1.peg.2094	73	
fig|1496.1.peg.2111	67	
fig|1496.1.peg.2183	30	
fig|1496.1.peg.2205	58	
fig|1496.1.peg.2274	78	
fig|1496.1.peg.2300	84	
fig|1496.1.peg.2349	78	
fig|1496.1.peg.2455	69	
fig|1496.1.peg.2490	87	
fig|1496.1.peg.2513	84	
fig|1496.1.peg.2546	96	
fig|1496.1.peg.2547	55	
fig|1496.1.peg.2551	98	Transposase

fig|1496.1.peg.2554	78	
fig|1496.1.peg.2577	95	
fig|1496.1.peg.2633	63	
fig|1496.1.peg.2634	75	
fig|1496.1.peg.2638	37	
fig|1496.1.peg.2737	59	
fig|1496.1.peg.2738	70	
fig|1496.1.peg.2774	44	MSL leader peptide 2
fig|1496.1.peg.2776	46	Omega protein
fig|1496.1.peg.2778	44	MSL leader peptide 2
fig|1496.1.peg.2779	62	hypothetical protein
fig|1496.1.peg.2804	64	
fig|1496.1.peg.2849	47	
fig|1496.1.peg.2859	93	
fig|1496.1.peg.2873	93	
fig|1496.1.peg.2899	68	
fig|1496.1.peg.2971	355	3-dehydroquinate synthase (EC 4.2.3.4)
fig|1496.1.peg.3011	67	
fig|1496.1.peg.3023	59	
fig|1496.1.peg.3193	94	
fig|1496.1.peg.3204	65	
fig|1496.1.peg.3228	33	
fig|1496.1.peg.3234	182	
fig|1496.1.peg.3236	34	
fig|1496.1.peg.3240	269	Methyltransferase
fig|1496.1.peg.3241	80	
fig|1496.1.peg.3242	65	
fig|1496.1.peg.3244	110	hypothetical protein
fig|1496.1.peg.3246	242	sulfate adenylate transferase, subunit 2 (cysD)

fig|1496.1.peg.3375	70	
fig|1496.1.peg.3399	61	
fig|1496.1.peg.3467	69	
fig|1496.1.peg.3513	62	
fig|1496.1.peg.3537	36	
fig|1496.1.peg.3538	34	
fig|1496.1.peg.3539	28	
fig|1496.1.peg.3654	35	
fig|1496.1.peg.3675	66	
fig|1496.1.peg.3727	97	
fig|1496.1.peg.3776	77	hypothetical protein
fig|1496.1.peg.3787	68	
fig|1496.1.peg.3886	80	
fig|1496.1.peg.3906	70	
fig|1496.1.peg.3922	190	
fig|1496.1.peg.3923	187	hypothetical protein
fig|1496.1.peg.3924	61	
fig|1496.1.peg.3925	64	
fig|1496.1.peg.3934	55	
fig|1496.1.peg.3936	108	hypothetical protein
